# Epithelial Cell Polarity During *Drosophila* Midgut Development

**DOI:** 10.3389/fcell.2022.886773

**Published:** 2022-06-30

**Authors:** Jia Chen, Daniel St Johnston

**Affiliations:** Gurdon Institute and the Department of Genetics, University of Cambridge, Cambridge, United Kingdom

**Keywords:** *Drosophila*, midgut, polarity, apical, basal, junction

## Abstract

The adult *Drosophila* midgut epithelium is derived from a group of stem cells called adult midgut precursors (AMPs) that are specified during the migration of the endoderm in early embryogenesis. AMPs are maintained and expanded in AMP nests that lie on the basal side of the larval midgut throughout the larval development. During metamorphosis, the larval midgut undergoes histolysis and programmed cell death, while the central cells in the AMP nests form the future adult midgut and the peripheral cells form the transient pupal midgut. Here we review what is known about how cells polarise in the embryonic, larval, pupal and adult midgut, and discuss the open questions about the mechanisms that control the changes in cell arrangements, cell shape and cell polarity during midgut development.

## Introduction

The *Drosophila* intestine is composed of several different cell types, including epithelial cells, muscle cells, neurons, and trachea cells (Miguel-Aliaga et al., 2018). The gut tube is formed by single layer of polarised epithelial cells surrounded basally by the muscles, trachea and nerves. The fly intestine is anatomically organised into the foregut, midgut and hindgut regions with the crop and Malpighian tubules emanating at the foregut/midgut and hindgut/midgut boundaries. The midgut is the longest section of the intestine and forms the conduit between the foregut and hindgut. It can be further subdivided into the anterior, middle and posterior midgut, which are marked by tissue constrictions and differences in luminal pH (Shanbhag and Tripathi, 2005, 2009). The epithelium performs the major functions of the midgut: acting as a barrier between the gut lumen and the inside of the organism and absorbing nutrients. It is composed of two types of mature epithelial cells: enterocytes (EC) and enteroendocrine (ee) cells. Based on cell morphology, physiology and gene expression profiles, epithelial cells in the midgut can be further classified into at least 10 different subregions and 22 clusters (Buchon et al., 2013; Marianes and Spradling, 2013; Dutta et al., 2015; Hung et al., 2020).

**FIGURE 1:**
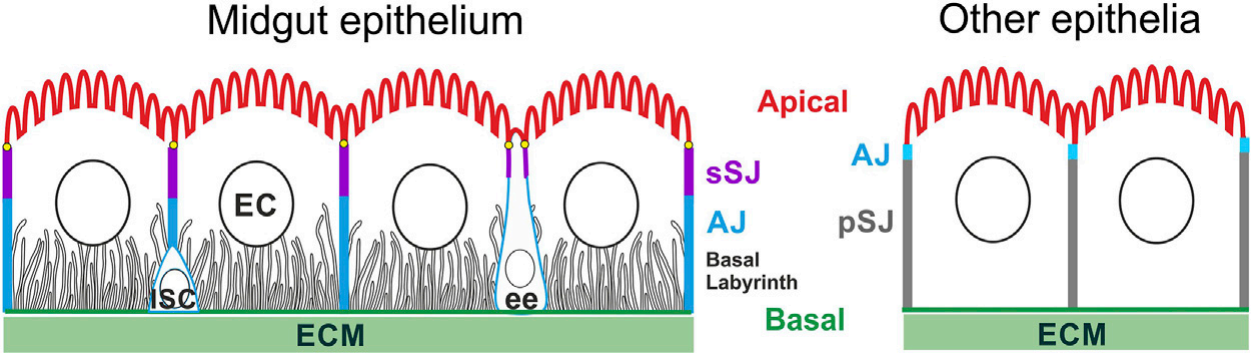
The apical-basal organisation of the *Drosophila* adult midgut epithelium in comparison with other epithelia. Intestinal stem cells (ISC) can differentiate into enterocytes (EC) and enteroendocrine cells (ee). The apical domain forms a brush border facing the gut lumen; the basal membrane contacts the ECM and develops long invaginations that form the basal labyrinth. The lateral domain contains apical smooth septate junctions (sSJ), above lateral adherens junctions (AJ). By contrast, the AJs form apical to the pleated SJs in the other *Drosophila* epithelia. The ee cells typically adopt a bottle shape with the cell body shifted basally, and a narrow neck ending with a bulbous apical domain facing the gut lumen.

Despite their diverse shapes and gene expression profiles, all epithelial cells share the same features of apical-basal polarity along the whole midgut (Figure 1). The major type of epithelial cells in midgut, the ECs, are absorptive and are usually of cuboidal/columnar shape, but in the middle midgut, specialised acid secreting ECs called the copper cells adopt a cup shape (Hoppler and Bienz, 1994; Strand and Micchelli, 2011). The apical membrane of ECs is covered in a brush border of microvilli, while infoldings of the basal membrane generate the basal labyrinth. Each structure serves to maximize surface area, which is thought to facilitate nutrient absorption, although how the basal labyrinth forms and functions is not well studied (Shanbhag and Tripathi, 2009; Sauvanet et al., 2015). The ee cells are secretory cells that release neuropeptide hormones in response to gut contents (Beehler-Evans and Micchelli, 2015; Hung et al., 2020). They exist as isolated diploid cells throughout the midgut epithelium, often have a bottle or fusiform shape and have little or no basal labyrinth. Recent work has shown that ee cells lack an apical brush border (Xu et al., 2018), as observed in midgut endocrine cells in other insect species (Billingsley and Lehane, 1996).

The brush border is enriched in actin and contains MyoIA, MyoIB and other actin crosslinking proteins (Table 1) (Morgan et al., 1995; Crawley et al., 2014). The region of the cortex at the base of the microvilli, which is called the “terminal web” in mammalian cells, contains MyoIA, Myo7a, aPKC and Par-6 (Table 1) (Morgan et al., 1995; Chen et al., 2018). The apical domain is supported by a submembraneous spectrin scaffold composed of β_H-_spectrin/α-spectrin heterotetramers that links the membrane to the actin cytoskeleton (Table 1) (Baumann, 2001; Chen and St Johnston, 2022). The apical surface of the epithelial layer in the adult is closely associated with, but does not necessarily contact, the peritrophic membrane (PM), which is composed of Type I PM produced by the entire midgut and type II PM that is secreted by the cardia/proventriculus at the most anterior region in midgut (Lehane, 1997). The PM serves a similar function to the mucous lining in mammalian gut as the outmost protective barrier (Zhang et al., 2017).

**TABLE 1:** *Drosophila* genes and their encoded protein’s localization during midgut development.

*Drosophila* gene (Abbreviation)/Alias	Human ortholog	Protein type	Protein localisation in the *Drosophila* midgut epithelial cell	References
Myosin 31DF (Myo31DF)/MyoIA	*MYOID*	Myosin	Apical brush border and terminal web in stage 17 E^#1^, L^#2^ and A^#3^	Morgan et al. (1995); Crawley et al. (2014)
Myosin 61F (Myo61F)/MyoIB	*MYOIC*		Relocates from basolateral domain to apical brush border in stage 17 E; apical brush border in L and A	
Crinkled (ck)/myosin VIIA (myo7a)	*MYO7A*		Apical in A	Chen et al. (2018)
Atypical protein kinase C (aPKC)	*PRKCI/PRKCZ*	Kinase		
Par-6	*PARD6*	PDZ^#4^		
Bazooka (baz)/par-3	*PARD3*		Apical side of the lateral junction in stage 9 E	Campbell et al. (2011)
Karst (kst)/β_Heavy_-spectrin	*SPTBN5*	Spectrin	Apical domain in L and A	Baumann, (2001); Chen et al. (2018)
β Spectrin (β-Spec)	*SPTBN1*		Basolateral domain in L and A	
α Spectrin (αSpec)	*SPTAN1*		Cell cortex in L and A	
Cheerio (cher)	*FLNA*	Actin cross linker, filamin	Apical in A; basal in stage 12/13 E	Chen and St Johnston, (2022);
				Devenport and Brown, (2004)
Crumbs (crb)	*CRB1*	TM^#5^	Apical in stage 9 E	Campbell et al. (2011)
Stardust (sdt)/pals1	*MPP5*	PDZ		
Stranded at second (sas)	*-*	TM		
Rhea/talin	*TLN*	FERM^#6^	Basal domain in stage 12 E and A	Devenport and Brown, (2004); Chen et al. (2018)
Fermitin 1 (Fit1)	*FERMT*		Basal domain in A	
Fermitin 2 (Fit2)	*/KINDLIN*			
Integrin linked kinase (Ilk)	*ILK*	Kinase		
Multiple edematous wings (mew)/αPS1	*ITGA6/7*	TM, ECM receptor	Mainly basal in stage 12–15 E; basal in A	(Yee and Hynes, 1993; Martin-Bermudo et al., 1999; Lin et al., 2013; Okumura et al., 2014; Pitsidianaki et al., 2021)
Inflated (if)/αPS2	*ITGA8*		Muscle layer	
Scab (scb)/αPS3	*ITGA4*		Mainly apical in stage 12–15 E; basal in A	
Myospheroid (mys)/βPS	*ITGB1*		Mainly basal in E; basal in A
Integrin betanu subunit (Itgbn)/βν	-		Mainly apical in E; basal in A
Frazzled (fra)/DCC	*NEO1*		Basal domain in from stage 12 E	Pert et al. (2015)
Dystroglycan (Dg)	*DAG1*		Tissue constriction region in stage 16 E	Schneider and Baumgartner, (2008)
Division abnormally delayed (dally)	*GPC5*	Glypican TM	-	-
Dally-like (dlp)	*GPC4*		-	-
Syndecan (Sdc)	*SDC*	Proteo-glycan TM	-	-
Laminin A (LanA)	*LAMA5*	ECM	LanA heterotrimer is mainly basal between the endoderm and mesoderm, also surrounding ICP cells and weakly at apical side in E; basal in L and A	(Wolfstetter and Holz, 2012; Lin et al., 2013; You et al., 2014; Pert et al., 2015; Töpfer and Holz, 2020; Pitsidianaki et al., 2021)
Wing blister (wb)	*LAMA1*		Basal ECM in E, L and A	
LanB1/LamininB1	*LAMB2*			
Laminin B2 (LanB2)	*LAMB2*		
Collagen type IV alpha 1 (Col4α1)/Cg25C	*COL4A1*		Basal ECM from stage 16 E, L and A
Viking (Vkg)	*COL4A1*		
Terribly reduced optic lobes (trol)/Perlecan	*HSPG*			
Nidogen (Ndg)	*NID1*		Basal ECM from stage 16 E and L	
Netrin-A (NetA)	*NTN1*		Basal ECM from stage 12 E	
Netrin-B (NetB)				
Secreted protein, acidic, cysteine-rich (SPARC)	*SPARC*		Basal ECM from stage 16 E and L	
Macrophage derived proteoglycan-1 (Mdp-1)/papilin (ppn)	-			
Glutactin (Glt)	-		Basal ECM in E	Olson et al. (1990)
Peroxidasin (Pxn)	*PXDN*			Nelson et al. (1994)
Mesh	*SUSD2*	TM	SJs from stage 16 E, L and A	(Izumi et al., 2012, 2016, 2021)
Snakeskin (Ssk)	-			
Tetraspanin 2A (Tsp2A)	*TSPAN8*			
Hoka	-			
Bark beetle (bark)/anakonda (aka)	-	TM	Tri-cellular junctions in E	Byri et al. (2015);
Gliotactin (Gli)	-			Wittek et al. (2020)
M6	*GPM6A*			
Shotgun (shg)/DECad	*CDH20*	TM Cadherin	Apical side of the lateral junction in stage 9 E; AJ in A	Campbell et al. (2011); Chen et al. (2018)
armadillo (arm)/β-catenin	*CTNNB1*	Armadillo repeat	AJ in A	Chen et al. (2018)
α Catenin (α-Cat)	*CTNNA*	Catenin		
Discs large 1 (dlg1)	*DLG1*	PDZ	Apical side of the lateral domain in the developing adult midgut at pupal stage	Takashima et al. (2011b)
Fasciclin 3 (Fas3)	*NECTIN3*	TM		

-, Not found.

#1,2,3 E, L and A denote the embryonic, larval and adult midgut epithelium separately.

#4, PDZ domain containing scaffolding protein.

#5, TM denotes transmembrane protein.

#6, FERM domain containing protein.

As mentioned above, the basal sides of the epithelial cells contact the extracellular matrix (ECM), except for the invaginations of the basal labyrinth, which do not appear to have any ECM in their lumens (Baumann, 2001; Shanbhag and Tripathi, 2009). Integrin associated proteins, such as Integrin linked kinase (Ilk), Rhea and Fit localise to the basal cortex (Table 1) (Chen et al., 2018). The ECM is assembled into a sheet-like basement membrane (BM) between the epithelium and the visceral muscle layers (Shanbhag and Tripathi, 2009). All four main types of the basement membrane components are present: type IV collagen (α1_2_α2 heterotrimers with Col4a1 as the α1 subunit and Vkg as the α2 subunit), Laminins (αβγ-heterotrimers with LanA and Wb as α subunits, LanB1 as the β subunit and LanB2 as the γ subunit), Nidogen and Perlecan (Table 1) (Broadie et al., 2011; Davis et al., 2019; Töpfer and Holz, 2020). Laminins and Type IV collagen form independent mesh-like structures with the Laminins closer to the epithelial cells. In addition, the gut BM contains Netrins (Pert et al., 2015), Secreted protein, acidic, cysteine-rich (SPARC) (Martinek et al., 2002, 2008), Macrophage derived proteoglycan-1 (MDP-1) (Kramerova et al., 2003), Glutactin (Olson et al., 1990) and Peroxidasin (Table 1) (Nelson et al., 1994).

Unlike most other fly epithelia, the *Drosophila* midgut epithelium is derived from the endoderm and the intercellular junctions in both the EC and ee cells have a different morphology and arrangement from the junctions in non-endodermal epithelia (Figure 1). Endodermal epithelia form smooth septate junctions (sSJs), analogous to tight junction in mammals, which lie apical to the adherens junctions (AJs), whereas in other epithelial cells, the electron-dense AJs lie above the septate junctions, which are pleated not smooth (Figure 1) (Lane and Skaer, 1980; Tepass and Hartenstein, 1994b; Baumann, 2001). Recent studies reveal that the smooth SJs are organised by the endoderm-specific proteins, Mesh, Snakeskin, Tsp2a and Hoka, which form a transmembrane protein complex (Table 1) (Izumi et al., 2012, 2016, 2021; Furuse and Izumi, 2017). The SJs at the vertices where three cells meet contain additional components, including Bark, Gli and M6, which are also found in the tri-cellular junctions in epithelia with pleated SJs (Table 1) (Schulte et al., 2003; Byri et al., 2015; Hildebrandt et al., 2015; Bosveld et al., 2018; Esmangart de Bournonville and le Borgne, 2020; Wittek et al., 2020). Loss of these tri-cellular SJ proteins during ageing leads to defects in the function of the intestinal barrier in older flies (Resnik-Docampo et al., 2017). In ectodermally-derived epithelia, Sidekick localsies to the tri-cellular AJs and modulates apical adhesion and tension during the active junctional remodelling during embryo morphogenesis (Finegan et al., 2019; Letizia et al., 2019; Uechi and Kuranaga, 2019). It is not known whether bi- or tri-cellular AJ in the midgut also contain specific components since ECad, Arm and α-Cat are the only known components of AJs in the midgut (Choi et al., 2011; Campbell and Casanova, 2015; Liang et al., 2017).

During the past 20 years, *Drosophila* midgut has proven an exciting model system to study epithelial homeostasis, since basally-localised intestinal stem cells (ISC) can divide and differentiate into both ECs and ee cells in the adult midgut. The signals and mechanical cues that regulate ISC division and differentiation have been extensively characterised and have been summarised in many excellent reviews of this topic (Micchelli and Perrimon, 2006; Ohlstein and Spradling, 2006; Jiang and Edgar, 2011; Lucchetta and Ohlstein, 2012; Zeng et al., 2013; Antonello et al., 2015; He et al., 2018; Miguel-Aliaga et al., 2018; Reiff and Antonello, 2019; Rojas Villa et al., 2019; Jasper, 2020). The ISCs reside beneath the tri-cellular SJs between ECs, and do not contact the gut lumen or have an apical brush border, forming only AJs with their neighbours (Shanbhag and Tripathi, 2009; Xu et al., 2018; Chen and St Johnston, 2022). ISCs divisions give rise to new ISCs and to enteroblasts (EBs), which are the post-mitotic precursor of the ECs. EBs remain quiescent until new ECs are required, either through damage or normal cellular turnover. They are activated to differentiate into ECs by a network of transcription factors, including Zfh2, Sox100B and Sox21a (Meng and Biteau, 2015; Zhai et al., 2015, 2017; Chen et al., 2016; Doupé et al., 2018; Rojas Villa et al., 2019). Once activated, differentiating EBs polarise as they integrate into the epithelium (Chen and St Johnston, 2022; Moreno-Roman et al., 2022) (note #1). When the EB reaches the SJ between the overlying ECs, ECad containing AJs are cleared from its apical surface. The margins of the apical surface form new SJs with the neighbouring ECs and the centre becomes an apical membrane initiation site (AMIS). Secretion of apical components at the AMIS then leads to the formation of a preformed apical compartment (PAC) with a brush border beneath an intra-epithelial lumen that forms below the overlying EC-EC septate junction. As the differentiating EB/pre-EC expands further apically, the EC-EC SJ disassembles from its basal side and it eventually disappears when the EB/pre-EC reaches the gut lumen. It is not known how the ee precursor cells differentiate and integrate into the epithelia layer, although early work described a “closed” type of ee cell identified by the electron dense secretory granules in midguts of other insect species. These cells do not contact the apical lumen, have minimal basal contacts with the basement membrane and may represent an intermediate stage in ee cell differentiation (Billingsley and Lehane, 1996; Caccia et al., 2019).

The exact mechanism that polarises ECs and ee cells in the adult midgut is not understood, but this does not require any of the canonical epithelial polarity factors that polarise non-endodermal epithelia, including Bazooka (Par-3), Par-6, atypical protein kinase C (aPKC), Crumbs (Crb), Stardust (Sdt), Discs large (Dlg), Lethal (2) giant larvae (Lgl) or Scribble (Chen et al., 2018). Instead, the basally localised integrin associated proteins, Rhea and Fit1 are required for all steps in EC polarisation and sSJ components are required for the formation of the PAC during EB integration (Chen et al., 2018; Chen and St Johnston, 2022). The progenitor and precursor cells, ISCs and EBs, lie at the basal side of the epithelium without any access to the apical lumen and do not form SJs with neighbouring cells. This indicates that the midgut epithelial cells require sustained basal signalling from the contact with the ECM to polarise in a basal to apical fashion. Their further polarisation, including the formation of sSJ and the apical brush border, requires positional cues from the SJs and the gradual growth of the apical domain via polarised membrane trafficking. The *Drosophila* midgut epithelium provides an excellent model for mammalian epithelia, which have a similar junctional arrangement and also require ECM contacts for polarity (Yu et al., 2005).

The adult *Drosophila* midgut epithelium is derived from AMPs, which are specified during early embryogenesis and segregated from the cells of the larval and pupal midgut during development (Campos-Ortega and Hartenstein, 1985; Tepass and Hartenstein, 1994a, 1995; Takashima et al., 2011b, 2011a, 2016a). Developmentally, the midgut epithelium is categorised as “a secondary epithelium”, since it goes through an epithelial-to-mesenchymal-transition (EMT) during early endoderm formation and later undergoes a mesenchymal-to-epithelial-transition (MET) to repolarise. In embryos, both the migration of the midgut primordia and repolarisation require basal contact with the mesoderm and ECM components surrounding the endoderm layer (Tepass and Hartenstein, 1994a; Yarnitzky and Volk, 1995). It has been suggested that a similar mechanism is deployed during EB polarisation and differentiation, when EBs acquire a migratory potential before repolarising and integrating into the epithelial layer (Micchelli, 2012; Antonello et al., 2015). In this review, we will describe what is known about cell polarity changes during embryonic, larval and pupal midgut development and discuss what this suggests about the mechanisms of apical-basal polarisation in endodermal tissues.

### Cell Polarity During Embryonic Midgut Development

**FIGURE 2:**
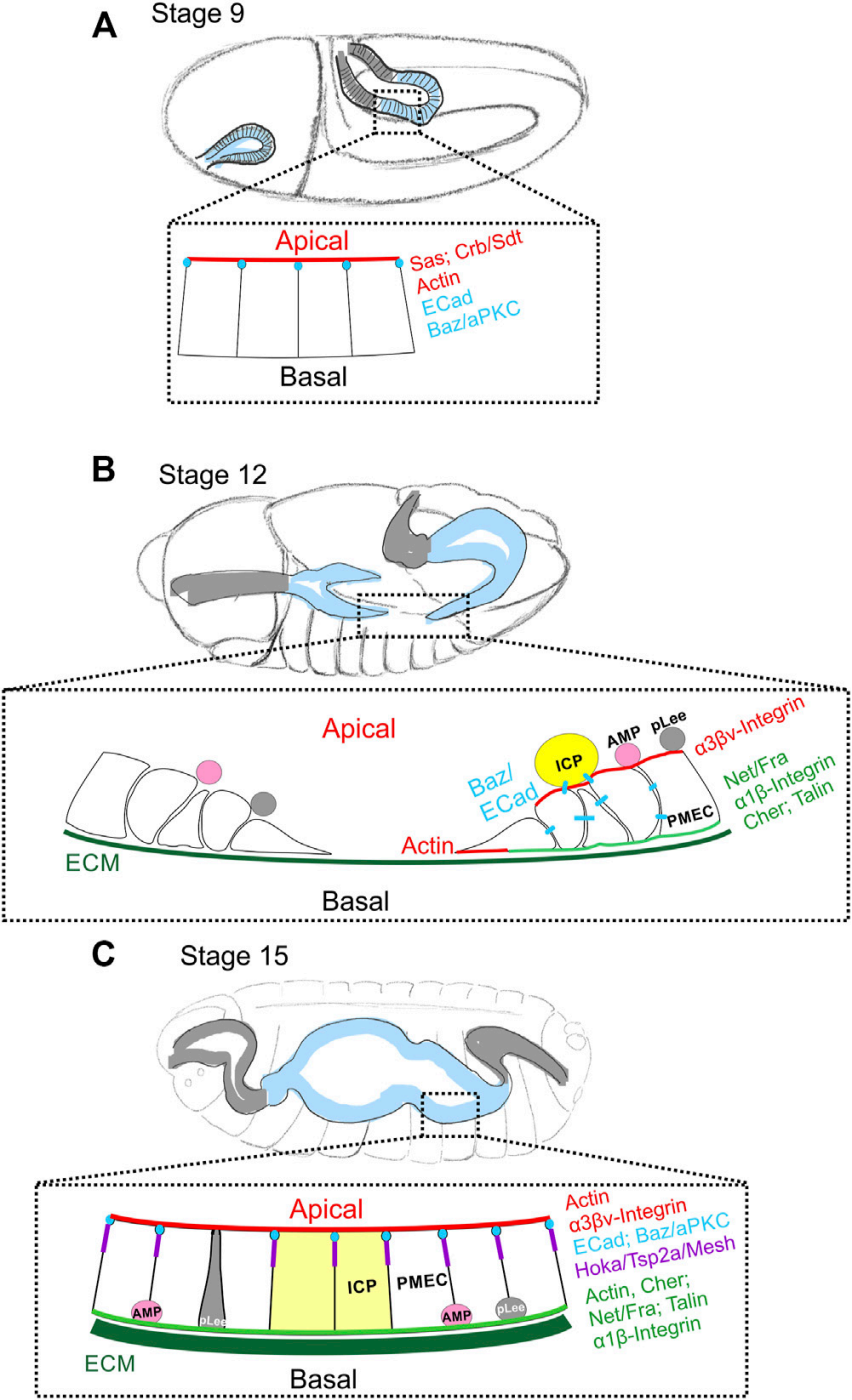
Changes in cell polarity during embryonic midgut formation. **(A)** During gastrulation at stage 9, the endoderm (blue shaded region) and ectoderm of the hindgut (gray shaded region) invaginate. The posterior midgut (all *esg*+) is still an epithelium with Sas and Crb/Sdt at the apical domain (red) and Ecad and Baz/aPKC at the apical AJs (blue). At this stage, the visceral muscle layer is not yet fully formed, and no clear basal features have been described. **(B)** By stage 12, Crb/Sdt and Sas have disappeared from the midgut primordia and the cells have undergone EMT and become migratory. The presumptive posterior and anterior midgut rudiments migrate along the visceral mesoderm towards each other. ECM (dark green) components can be found between the endoderm and visceral mesoderm by late stage 12. The posterior midgut primordium segregates into principal midgut epithelial cells (PMECs), Interstitial cell precursors (ICPs; yellow) and adult midgut precursors (AMPs; pink), while the anterior primordium contains only PMECs and AMPs. ICPs to delaminate first, followed by the AMPs and both remain attached to the migrating PMECs. At stage 11, the inner layer of migrating mesenchyme also contains *esg* + Pros + cells, possibly the progenitors of the larval ee cells (pLees; gray). Both the AMPs and pLees remain attached to PMECs until later stages. Actin is enriched at the basal, migratory front and Baz (blue) can be found at spot AJs between PMECs and ICPs. Behind the migrating front, PMEC cells start to repolarise. Talin and Filamin1/Cher are localised basally (green) together with Fra and the α1/β-integrin complex, while the α3βν-integrin complex localises apically (red). **(C)** By stage 15, the anterior and posterior midgut primordia have fused and the presumptive midgut has closed ventrally and dorsally to form a continuous tube. ECM (dark green) forms a more complex network at this stage. The repolarised PMECs start to form smooth SJs (purple). ECad and Baz localise to the apical junctions (blue), Actin to both the apical and basal sides and Filamin-1/Cher to the basal domain. Fra and the α1β-integrin complex remain at the basal domain (green), while the α3βν-integrin complex localises mainly apically (red). By the end of embryonic development, ICPs (yellow) have integrated into larval midgut epithelium and AMPs (pink), which are the only remaining *esg* + cells, have translocated to the basal side of the epithelium. It is not known when the pLee cells (grey) integrate into the epithelium.

The *Drosophila* midgut primordium forms from the endoderm during gastrulation (Campos-Ortega and Hartenstein, 1985). Under the coordinated action of the GATA transcription factor Serpent and the winged-helix transcription factor Forkhead, the posterior midgut primordium (PMG) together with the ectodermally-derived hindgut primordium are internalised into the embryo (Weigel et al., 1989; Reuter, 1994; Nakagoshi, 2005). The PMG cells initially have the same apical-basal polarity as all ectodermal cells, which is established during the process of cellularisation (Tepass and Hartenstein, 1994b). Stranded-at-second (Sas), and the canonical apical polarity factors, Crb and Sdt, localise to the apical surface and Baz and ECad are localised to the apical AJ (Table 1) (Figure 2A) (Campbell et al., 2011). During stage 10 of embryogenesis, Serpent induces the PMG to undergo an EMT and become migratory by repressing the expression of Crb, Sdt, Sas, and pleated SJ genes (Tepass and Hartenstein, 1994b; Campbell et al., 2011). As a result, the apical AJs dissolve and ECad and Baz relocalise from the AJs to dynamic puncta at cell-cell contacts, which are presumably scattered spot AJs. At stage 11, the PMG has established contact with the visceral muscle primordium and uses it as a substrate for its migration (Tepass and Hartenstein, 1994a). Three different cell types can be distinguished transcriptionally and morphologically among the migrating midgut mass. Most cells are principal midgut epithelial cells (PMECs), which will give rise to the larval midgut ECs and always contact the muscle primordium. The other two populations of mesenchymal cells, interstitial cell precursors (ICPs) and AMPs, are attached to the apical surface of PMECs and are carried along by the latter. ICPs express Inscuteable and Asense from late stage 10 to mid-stage 11, and AMPs, which will give rise to the future adult midgut, are Asense-positive from early stage 11 to late stage 12 (Figure 2B) (Tepass and Hartenstein, 1994a, 1995; Campbell and Casanova, 2015). ICPs and AMPs delaminate sequentially from the outer layer of PMECs between stage 10–11 during their posterior migration (Tepass and Hartenstein, 1995). Some AMPs at this stage can also be marked with anti-Pros antibody staining, suggesting that they may be progenitor cells for future larval ee cells (pLee), although there is no lineage tracing data to support this. Like AMPs, pLees remain in the mesenchymal inner mass during migration, but become *esg*- and segregate from the AMPs by stage 14 (Jiang and Edgar, 2009; Takashima et al., 2011a). The cohesive and ordered migration of these three/four types of cells along the visceral mesoderm is coordinated through ECad-mediated cell adhesion and relies on the Integrin/Laminin and Frazzled/Netrin signalling pathways (Martin-Bermudo et al., 1999; Devenport and Brown, 2004; Campbell and Casanova, 2015; Pert et al., 2015; Pitsidianaki et al., 2021). Between late stage 11 and stage 12, shortly before and during germ band retraction, the PMECs reorganize and go through MET to form the midgut epithelium. By the end of germ band retraction at stage 13, the anterior and posterior midgut rudiments approach each other and finally fuse, the PMECs assume a columnar shape and the ICPs form two clusters in the middle of the developing midgut (Tepass and Hartenstein, 1994a). MET coincides with the downregulation of Fkh and Srp (Weigel et al., 1989; Campbell et al., 2011). However, Srp down-regulation is not sufficient to trigger MET, which instead depends on basal cues from Laminin and Netrins produced by the visceral mesoderm acting through Integrins and Fra respectively (Pert et al., 2015; Pitsidianaki et al., 2021) (Figure 2B and discussed later).

During endoderm migration, the ECM between the endoderm and the mesoderm is not yet fully organised, since early electron microscopy studies demonstrated that PMEC migration is mediated through direct mesoderm/endoderm contact without any detectable ECM or junctional specialisations (Tepass and Hartenstein, 1994a). However, Srp activates *LanB1* and *LanB2* RNA expression in stage 11 midgut primordium cells (Wolfstetter and Holz, 2012; Töpfer et al., 2019). Moreover, the laminin matrix secreted by the visceral muscle primordium contains Wb, which is thought to induce MET, whereas that secreted by endodermal cells contain LanA, and both LanA and Wb play crucial roles in controlling the speed of migration (Urbano et al., 2009; Wolfstetter and Holz, 2012; Pitsidianaki et al., 2021). At this stage, haemocytes (migrating macrophages) are the only source of secreted type IV collagen and Perlecan (Matsubayashi et al., 2017) and they do not reach the endoderm until after the migration is complete (Urbano et al., 2011; Pitsidianaki et al., 2021). Nidogen is reported to have similar expression pattern to LanB1 during embryogenesis but is not required for endoderm migration or formation (Urbano et al., 2009; Dai et al., 2018; Töpfer and Holz, 2020). At stage 16, Laminins, Collagens, Nidogen, and Perlecan, as well as other mature ECM components, such as MDP-1 and SPARC are all found in between the endoderm and mesoderm, forming a more complex ECM network (Wolfstetter and Holz, 2012).

Cells rely on ECM receptors to receive migratory/adhesive cues from the ECM, including Integrins, Fra, Dystroglycan (Dg), the Glycipans Dally and Dally-like and Syndecan (Sdc) (Table 1). Integrins function as heterodimers of α and β subunits and are required for both midgut migration and visceral muscle formation (Devenport and Brown, 2004). Flies have five α integrin subunits, αPS1-5 and two β subunits, Mys and βν (Table 1). Embryonic midgut migration requires the expression of both αPS1 in the endoderm and αPS2 in the visceral muscle, while αPS3 cooperates with αPS1 in the endoderm layer but is not required (Brown, 1994; Stark et al., 1997; Martin-Bermudo and Brown, 1999; Martin-Bermudo et al., 1999). Phylogenetic studies show that the αPS3-5 subunits are closely related and the result of gene duplication events (Hughes, 2001). αPS3 and αPS4 are expressed in adult midgut ECs, whereas αPS5 is not (Lin et al., 2013; Patel et al., 2015). Mys is widely expressed and is essential for viability, whereas βν is specifically expressed in the developing endoderm and the larval and adult midgut, but is not required for viability or fertility (Yee and Hynes, 1993). Integrins must form heterodimers in the endoplasmic reticulum to be trafficked to the cell surface and flies without both β subunits have no integrin function at all (Leptin et al., 1989; Devenport and Brown, 2004). Both αPS1/Mys and αPS3/βν pairs of integrins can be found in the migrating endoderm at late stage 11, with αPS1/Mys localising to basal side and αPS3/βν localising mainly apically at the end of migration (Figure 2B) (Devenport and Brown, 2004; Pitsidianaki et al., 2021). Two of the three Dystroglycan splicing isoforms are expressed in the midgut at stage 16, but their functions have not yet been characterised (Schneider and Baumgartner, 2008). Fra localises to the basal side of the PMECs at stage 12 and to the basal and junctional domain of the migrating midgut cells at stage 13 (Figures 2B,C). Interestingly, AMPs, which normally remain apical to the migrating PMECs at stage 12, are mis-localised and contact the visceral muscle in *netrin* mutant embryos. This phenotype has been attributed to the dis-organisation and loose adhesion of the PMG epithelium, rather than loss of direct signalling to AMPs, (Pert et al., 2015).

The PMECs are the first cell-type in the midgut primordia to go through MET, with AMPs, pLees, and ICPs remaining mesenchymal in the apical lumen until later. Although the exact time at which AMPs invade and translocate across the epithelium is not defined, they are located at the basal side of the gut in newly hatched larvae while the pLees have polarised and integrated into the epithelium (Hartenstein and Nung Jan 1992; Micchelli, 2012). This raises the question of how AMPs translocate to the basal side of the epithelium, since the apical junctions between the PMECs, which are marked by ECad, start to develop during migration in the outermost trailing region of the posterior midgut and sSJs start to develop in midgut from stage 15. Moreover, it is not clear whether the pLees become polarised and integrate into the epithelium during translocation or repolarise/integrate after translocating to the basal side (Takashima et al., 2011a). It has been hypothesized that the early delamination and late segregation and translocation of the AMPs and ICPs are due to differences in cell-cell affinity (Tepass and Hartenstein, 1995). However, there are no defects in the apical location of AMPs in *Ecad/shg* mutant embryos, it is therefore unclear whether their delamination and translocation is a passive cell-sorting event or an active migration process (Tepass and Hartenstein, 1994a). Furthermore, it will be important to determine the relationship between cell fate determination and the corresponding EMT-MET processes.

By stage 15, the visceral mesoderm (VM) expands ventrally and dorsally to form the circular muscle fibres and the endodermal layer follows this movement to form a closed chamber. Although the early specification of the endoderm into distinct PMEC, ICP, pLee and AMP cell types does not depend on interaction with the mesoderm, VM induces the further specification and development of future larval midgut epithelium after the midgut rudiments fuse, including the formation of the three midgut constrictions during stages 14–16 and the specification of the middle midgut region and proventriculus (Nakagoshi, 2005).

Between stage 16 and 17, the future larval ECs change their morphology from short cuboidal cells to tall columnar cells and develop elaborate cellular junctions and an apical brush border (Morgan et al., 1995). Smooth SJ components start to express during stage 12 and become localised at stage 16, but mature sSJs only become visible at late stage 17 (Tepass and Hartenstein, 1994a; Izumi et al., 2012). Myo61F relocates from the basal-lateral region to the apical microvilli, coincident with the disappearance of the yolk mass which indicates the start of digestive function (Morgan et al., 1995).

*Drosophila* embryonic midgut formation takes less than 9 h, between stage 10 when PMG starts EMT and stage 15 when the midgut migration finishes. Both EMT and MET happen gradually, whereas cell polarity changes dramatically and rapidly during this process. The apical polarity factor Crb disappears early on, the original apical-lateral junctions dissolve, giving rise to a group of mesenchymal migratory cells connected by limited spot AJs. These cells later re-polarise forming smooth SJs rather than pleated SJs at the apical/lateral side of the cell-cell junctions. Embryonic midgut development also demonstrates the importance of the sustained basal signalling from the mesoderm much like the polarisation of the EBs in the adult midgut epithelium. It is still unclear, however, what lies downstream of basal ECM and their receptors to induce epithelial polarisation, and whether apical extracellular LanA and apically localised αPS3/βν integrins plays any role in polarising the embryonic midgut epithelium. Past work has focused on the morphological development of the midgut and the genetic control of endoderm formation and differentiation (Bilder and Scott, 1995; Harbecke and Lengyel, 1995). Much less is known about the genetic control of AMP and ICP delamination and translocation, which also involves the loss and gain of cell polarity. These processes are challenging to study, however, because they occur over short time periods in the centre of the embryo.

### Larval Midgut Epithelial Cells

The larval midgut is composed of anterior, middle and posterior regions, each maintaining a different pH, and is anatomically similar to the adult midgut, although the constriction around the middle midgut is less obvious (Shanbhag and Tripathi, 2005; Overend et al., 2016). The larval midgut contains four gastric caeca, which are blind sacs that emerge from the anterior midgut just posterior to the proventriculus. They persist in the larva but are lost during pupation and are not present in the adult fly (Skaer, 1993). Larval ECs are polyploid and derive from PMECs, whereas larval ee (lee) cells are diploid and derive from pLees (Takashima et al., 2011a). Cell specification has been well-studied in the larval middle midgut (Hoppler and Bienz, 1994, 1995). Large cells in this region were first called calycocytes, and were later named cuprophilic or copper cells, since they accumulate copper and display orange fluorescence when the larvae are fed with copper-enriched food. This property is attributed to the binding of copper ions to metallothionein, which is constitutively expressed in the cytoplasm in the middle midgut region (Skaer, 1993; Durliat et al., 1995; McNulty et al., 2001). It has been proposed that copper cells derive from the ICPs, although this is at odds with the observation that the ICPs disseminate over the whole embryonic midgut after stage 15 (Poulson and Waterhouse, 1960; Skaer, 1993). The copper cells are cup-shaped, with an invaginated apical domain containing long microvilli. They are surrounded by columnar interstitial cells with a normal apical domain, short microvilli and a more extensive basal labyrinth (Filshie et al., 1971). It is thought that the copper cells are the acid secreting cells, based on the correlation between the number of residual copper cells in *labial* mutant larvae and the number of remaining acid-retaining cells (Hoppler and Bienz, 1994; Dubreuil et al., 1998; Dubreuil, 2004). Several V-ATPase and other ion transporters are required for the acidic pH generation (Overend et al., 2016; Tian et al., 2022). Interestingly, copper absorption from the food can inhibit acid secretion and the acid secretion defective *α-spec* mutant copper cells are not able to accumulate copper, which raises the question of how copper absorption and acid secretion are linked (Dubreuil et al., 1998; McNulty et al., 2001). Furthermore, we still do not know how and why the apical domain in the copper cells invaginates nor how the interdigitated arrangement of copper cells and interstitial cells arises. One clue comes from the stage 15 embryonic midgut, when the inner ICPs interdigitate between the outer *labial*-positive ICPs (Skaer, 1993), which means that the arrangement of copper cells and interstitial cells are probably also under the control of *labial*.

**FIGURE 3:**
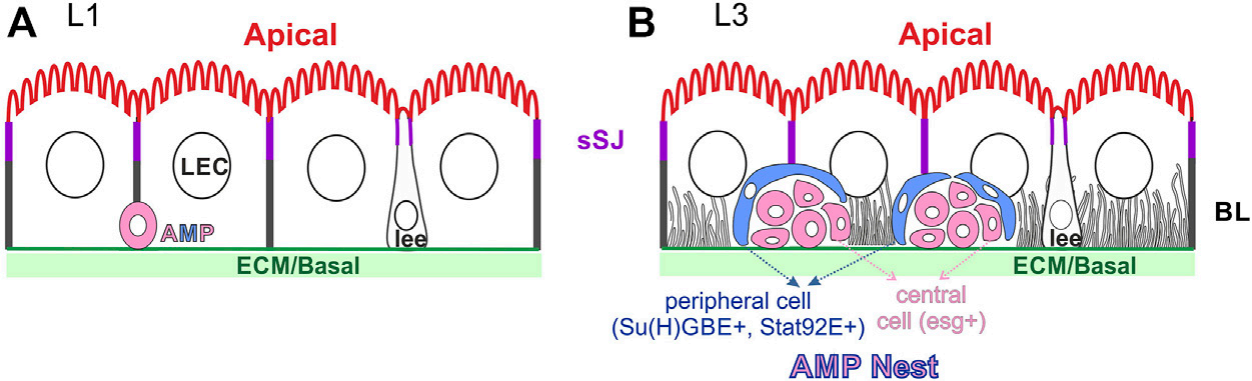
The organisation of the larval midgut. The larval enterocytes (LEC) have a similar polarity to the adult ECs, with an apical brush border, sSJs and a basal labyrinth (BL) (possibly only at later stages). The larval ee cells (lee) have inserted into the larval midgut epithelium and are bottle-shaped, like adult ee cells. **(A)** The *esg* + AMPs keep dividing during the first larval instar and the daughter cells migrate and distribute along the basal surface of the epithelium. **(B)** 1-3 *10xSTAT92E-GFP* + peripheral cells (blue) ensheath the diploid central, *esg* + cells (pink) to form the AMP nests in the late third instar larval midgut. Some cells in each nest become Pros + at this stage and will contribute to the future tPMG.

The larval midgut is remarkably similar to the adult midgut at the level of cellular structure, with an apical brush border facing the gut lumen, a basal side in contact with the visceral muscle and a basal labyrinth of invaginations from the basal membrane (Figure 3) (Shanbhag and Tripathi, 2005, 2009). The larval midgut also forms sSJs (Izumi et al., 2012, 2016, 2021) and the apical domain is enriched for actin and β_H-_spectrin/α-spectrin, while β-spectrin/α-spectrin heterotetramers label the basolateral domain (Dubreuil et al., 1998). Spectrins are not required for copper cell polarity, but loss of β_H-_spectrin leads to loss of the apical proton pump, the H^+^V-ATPase which probably causes the defect in acid secretion seen in *α-spec* mutant larvae (Phillips and Thomas, 2006). The two class I myosin family proteins, Myo31DF and Myo61DF can also be found in the apical terminal web and brush border microvilli in the larval ECs, but neither is required for cell polarity or brush border organisation (Morgan et al., 1995; Okumura et al., 2015). Interestingly, the AJs marked by ECad and Baz localise apical to the sSJ before the embryo hatches, whereas, AJs localise to the basal side of the sSJ in the first instar larva and adult ECs (Tepass and Hartenstein, 1994b; Chen et al., 2018). It is not clear how and when apical AJs disappear and basal AJs form during larval midgut formation.

One important feature of the larval midgut is the presence of AMPs. They first appear as single cells residing at the basal side of the larval midgut epithelium (L1, Figure 3A). They divide 7-10 times during the larval midgut development. The daughter cells of the first three divisions migrate and spread along the basal surface of the epithelium. The AMPs continue to divide during the third instar stage, but the daughter cells stay attached to each other to form AMP nests, which contain 8–30 cells by the onset of metamorphosis (Figure 3B) (Mathur et al., 2010; Takashima et al., 2011a; Jiang et al., 2011). 1-3 of the cells in an AMP nest differentiate into *STAT92E > GFP* (JAK-STAT pathway reporter) and *Su*(*H*)*GBE > GFP/lacZ* (Notch signalling reporter)-positive peripheral cells, which elongate and surround the inner mass of small, round central cells. Peripheral cells are post-mitotic with bigger nuclei than the central cells. They function as a niche to maintain the stem-cell state of the central AMP cells until metamorphosis (Mathur et al., 2010). However, the central cells can differentiate and become partially regenerative when the larval midgut is challenged with infection (Houtz et al., 2019). Some AMP cells also differentiate into Pros + cells and become an integral part of the future transient pupal midgut (Takashima et al., 2011b). The AMP nests can reach 2/3 of the height of the larval ECs, but do not reach the apical lumen, presumably because they cannot pass the sSJs between the larval ECs (Figure 3B). Both peripheral cells and central cells appear to maintain the contact with ECM and the peripheral cells contact the larval ECs. It is not known how the peripheral cells adopt a sheath-like shape and encase the central cells, nor how they provide a niche for the central AMP cells, except that the Dpp signalling is required (Mathur et al., 2010).

### Pupal Midgut Epithelial Layer and the Formation of Adult Midgut Epithelium

**FIGURE 4:**
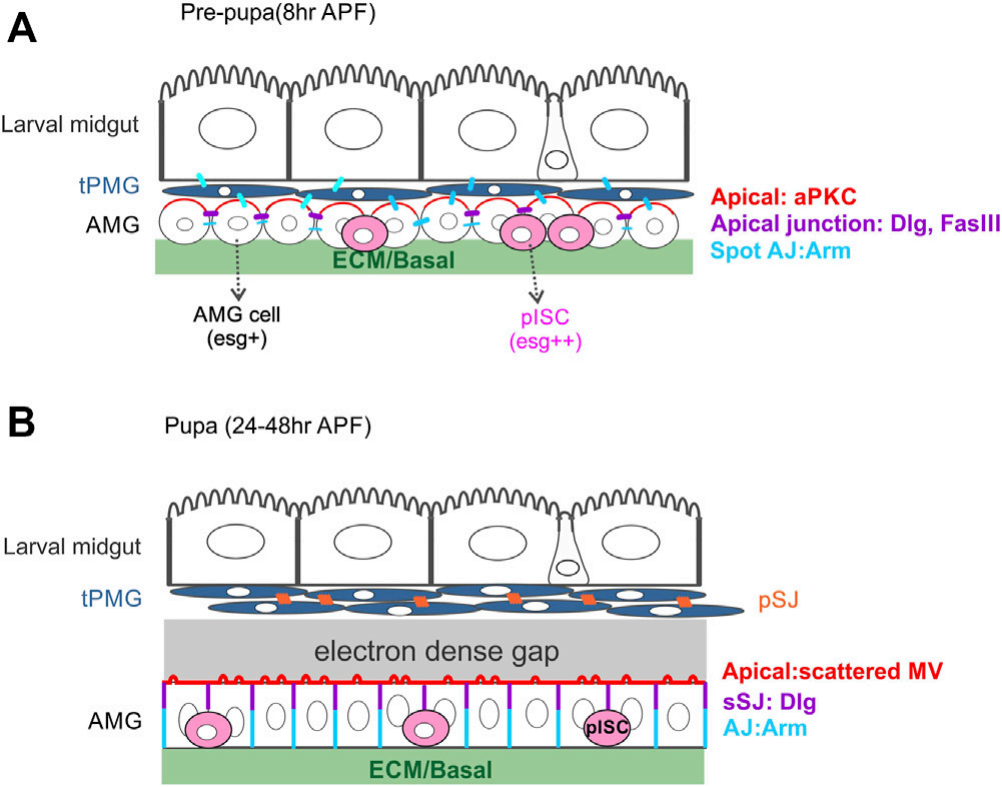
The organisation of the midgut during pupal development. **(A)** During the first hours after puparium formation, the peripheral cells of late larval midgut AMP nests re-arrange to form the tPMG (dark blue) around the degenerating larval midgut cells. At the onset of metamorphosis, the central cells of late larval midgut AMP nests spread out to surround the tPMG. This layer of AMP cells initially express *esg* homogenously, but most AMG cells downregulate *esg* as they differentiate into ECs. A subset of AMPs maintain *esg* expression and become the presumptive intestinal stem cells (pISCs, pink), the precursors of the adult intestinal stem cells. At this stage, aPKC (red) localises to the apical domain of the AMG cells and Dlg and FasIII to the apical side of the lateral domain (purple). Spot AJs (blue) connect the larval ECs, the tPMG and the AMG cells. **(B)** From 20 h APF onwards, the tPMG appears as a tightly packed multi-layered structure with pleated SJs (orange) connecting the cells. By this stage, the tPMG has separated from the surrounding AMG and an electron dense liquid can be found between the two tissues. The AMG starts to develop irregularly spaced apical microvilli and smooth SJs at the apical side of the lateral membrane. AJs connect the more basal regions of the lateral membrane. pISCs remain basally localised.

Shortly after puparium formation (APF), the larval midgut shortens, bringing the scattered AMP nests together. The outer peripheral cells contact each other first and by 6h APF become squamous and join together to form a multi-layered sheet called the transient pupal midgut (tPMG). Central cells also change their shape, flattening longitudinally and expanding laterally, to form a continuous layer of presumptive adult midgut (AMG) in a process that is thought to be MET (Takashima et al., 2011b). At this stage, the larval midgut, tPMG and AMG are all connected via spot AJs. At 8 h APF, the AMG starts to show polarised features, with aPKC localising apically, Fas3 at the apical and lateral domains and Arm along the lateral and basal domains. Precursors of the adult ISCs, the presumptive intestinal stem cells (pISCs), remain at the basal side of the AMG layer (Figure 4A). At the same time, the tPMG also differentiates to a certain degree, forming microvilli and containing ee cells that have a spindle shape and remain detectable until 24 h APF (Figure 4A, 3B) (Takashima et al., 2011b; Takashima et al., 2016b). At 6 h APF, the ECM layer surrounding the midgut and visceral muscle starts to break down and disappears by 24 h APF. By 36 h APF, myofibrils disappear since the visceral muscle fibres surrounding midgut de-differentiate into secondary myoblasts (Aghajanian et al., 2016). During this time, both the tPMG and the AMG keep differentiating. The tPMG develops pleated SJs, whereas the AMG develops apical microvilli and smooth SJs at the apical side of the lateral domain. The AMG is in direct contact with the myoblasts since no ECM is observed in between (Aghajanian et al., 2016). Interestingly, an electron dense liquid has been observed separating the larval and tPMG from the AMG at this stage (Figure 4B) (Takashima et al., 2011b). Both the myofibrils and ECM reorganise and reappear by 48 h APF (Aghajanian et al., 2016). Between 48 and 72 h APF, some esg-positive pISCs express Pros and divide asymmetrically to give rise to the adult ee cells (Guo and Ohlstein, 2015). The re-emergence of ECM is thought to be important for the pISC division and specification at this stage (Aghajanian et al., 2016). During later stages of metamorphosis, the larval and transient pupal midguts remain closely associated and further contract and become the “yellow body” in the lumen of the developing adult midgut. They are eventually discharged from the intestinal tract after eclosion.

The separation between larval midgut/tPMG and the AMG is essentially the delamination of larval midgut epithelial cells and the detachment between peripheral cells and central cells of the AMP nests. This results in the reorganisation of the tissue into three layers with spot AJ still present among them (Takashima et al., 2011b). The reorganisation happens within the first 12 h during pupal development, while the visceral muscle and ECM are still present. Both the tPMG and AMG keep differentiating, but only the AMG remains attached to the ECM, which means the separation cannot be simply explained by apoptosis-induced cell extrusion. It would be interesting to find out whether basal integrin adhesion is weakened in the larval epithelium and tPMG but retained in the AMG. Many other questions still remain about the adult midgut formation during pupal metamorphosis. First of all, before metamorphosis begins, there is direct signalling between the peripheral cells and central cells in the seemingly compact AMP nests, but almost nothing is known about the molecules that mediate adhesion between them or the molecular mechanisms that control the separation and reorganisation of the tPMG and AMG. Secondly, although the tPMG loses contact with the ECM and muscle layer, it still manages to differentiate to form pleated SJ. The functional significance of this junction and how it is formed are unclear. Thirdly, the AMG cells are believed to go through MET as they polarise, while the pISCs remain basal and in contact with the re-formed ECM and muscle layer. Based on what we know about the formation of the embryonic and adult midgut, it will be interesting to determine whether pISC specification requires a similar translocation process to AMPs in the embryo and if the AMG cells polarise in the same way as adult EBs and form a PAC as they integrate into the epithelium, and if their polarisation requires basal integrin signalling and SJ components.

## Concluding Remarks

AMPs are specified at an early embryonic stage, delaminate apically but remain attached to the PMECs via spot AJs and stay in the apical lumen of the migrating midgut primordium. They then translocate across the newly formed epithelium at the end of midgut development and remain basally after the embryo hatches. The mesoderm is not involved in the AMP specification and delamination, whereas cell-cell adhesion is proposed to play an important role in both the delamination and translocation. These processes are accompanied by the migration and repolarisation of the midgut primordium to form the future larval midgut epithelium. The migration and repolarisation require secreted Laminins from both germ layers, LanW at the basal side from the mesoderm and LanA at the apical side from the endoderm. The LanW from the basal side interacts with integrins receptors to activate downstream signaling pathways that are proposed to provide the cue that polarises the midgut epithelium and induce further polarised trafficking. The smooth SJs and the apical brush border microvilli form as the last step of polarisation in the epithelium. The polarised membrane features in the embryonic midgut epithelium are different from the steady state adult midgut epithelium, where integrin signalling components are only found basally and the lateral cell-cell junctions are clearly separated into apical-lateral sSJs and basal-lateral AJs. However, similar transcription factors control adult ISC maintenance and differentiation and embryonic midgut morphogenesis (Okumura et al., 2016). Moreover, EBs also go through a migratory stage before repolarising into ECs (Antonello et al., 2015). This means that the molecular mechanisms governing cell migration, cell translocation and MET-EMT could be the same in the embryo and adult.

During larval development, the AMPs expand, differentiate and form a nest containing peripheral cells and central cells. The peripheral cells are polarised to form sheath that surrounds and presumably isolates the central cells from the larval epithelial cells. It is not clear what type of cell-cell junctions form in the AMP nest and between the nest and larval epithelial cells. The peripheral cells later separate from the central cells to form the tPMG and delaminate with the larval epithelium at the start of pupation. By contrast, the central cells remain in contact with the basement membrane while adhering with each other to form the future AMG. Although both peripheral cells and central cells originate from AMPs, the peripheral cell-derived tPMG will develop pleated SJs instead of smooth SJs. During the separation and reorganisation, spot AJs are found connecting the larval midgut, tPMG and AMG. This raises the possibility that cell-cell adhesion dynamics regulate the separation. After the visceral muscle and ECM layer reform at the basal side, the central cells start to polarise. Little is known about how polarised domains form in the AMG, except that aPKC localises to the apical domain, Dlg and FasIII occupy the apical-lateral junction and Arm/Ecad are localised at the basal-lateral domain (Takashima et al., 2011b). Since both embryonic midgut formation and EB polarisation in the adult midgut require sustained basal signalling, it seems likely that the AMG requires basal signalling from the newly-formed ECM to polarise, but the molecular mechanisms remain to be discovered.

In summary, studies on the behaviour of stem cells and the stem cell niche in the *Drosophila* midgut during embryonic, larval, pupal and adult development have paved the way for investigations into how cells are specified at each stage and how their polarity is controlled. Elucidating the roles of cell-cell interactions and signals from the ECM in the control of cell fate, cell shape and cell polarisation, will advance our understanding of how the gut epithelium develops and functions under healthy conditions, and how this is perturbed in diseased states such as cancer.

## Note#1

By the time of submitting this review paper, these two research papers (Chen and St Johnston, 2022; Moreno-Roman et al., 2022) are still in the peer-reviewed stage for publishing. The citations are referring to the versions published on bioRxiv.org.

## Abbreviations

AJ, adherens junction; AMG, presumptive adult midgut; AMIS, apical membrane initiation site; AMP, adult midgut precursor; APF, after puparium formation; BM, basement membrane; EB, enteroblast; EC, enterocyte; ee, enteroendocrine cell; ECM, extracellular matrix; EMT, epithelial to mesenchymal transition; ICP, interstitial cell precursor; ISC, intestinal stem cell; MET, mesenchymal to epithelial transition; PAC, preformed apical compartment; pISC, presumptive intestinal stem cell; pLee, progenitor cell for larval enteroendocrine cell; PM, peritrophic membrane; PMEC, principal midgut epithelial cell; PMG, posterior midgut primordium; SJ, septate junction; tPMG, transient pupal midgut; VM, visceral mesoderm.
